# Enterocutaneous Fistula in a COVID-19 Obese Patient During Prolonged Prone Position for Severe Acute Respiratory Distress Syndrome

**DOI:** 10.7759/cureus.47212

**Published:** 2023-10-17

**Authors:** Nikitas Karavidas, Ismini Paraskeva, Georgios E Zakynthinos, Vasiliki Tsolaki

**Affiliations:** 1 Critical Care Medicine, University Hospital of Larissa, Larissa, GRC; 2 Surgery, University Hospital of Larissa, Larissa, GRC; 3 Cardiology, Athens Chest Disease Hospital "Sotiria", Athens, GRC

**Keywords:** septic shock [ss], sars-cov-2, acute respiratory syndrome, enterocutaneous fistulae, prone positioning

## Abstract

Prone position (PP) has been widely used in patients under mechanical ventilation for COVID-19 acute respiratory distress syndrome (ARDS), usually for many hours per day. Complications are not rare, although most of them are mild. To our knowledge, we report the first case of enterocutaneous fistula after prolonged use of PP in the literature. Morbid obesity; yielding increased abdominal wall pressure when the patient was prone; pre-existing intestinal hernias; and increased vasopressor doses for septic shock due to secondary infections resulted in necrosis of the small intestine, the abdominal wall, and the skin leading to enterocutaneous fistula. Clinicians managing patients with COVID-19 should keep in mind this complication, especially when proning obese patients with a history of intestinal surgery, as the presence of intestinal hernias might be missed during a clinical examination.

## Introduction

Severe acute respiratory distress syndrome by coronavirus-2 (SARS-CoV-2) infection primarily affects the respiratory system and can lead to the development of acute respiratory distress syndrome (ARDS), often necessitating invasive mechanical ventilation support [1]. Among various interventions, prone position (PP) has been extensively employed in ARDS patients during the COVID-19 pandemic. This approach has demonstrated improvements not only in oxygenation but also in the survival of the most severe patients (PaO2/FiO2 <100 mmHg), particularly among those with a consistent oxygenation response to PP [2-4]. Consequently, PP has been employed for extended durations and in multiple sessions during Intensive Care Unit (ICU) stays, especially in cases of refractory hypoxemia. However, the use of PP in ARDS has been linked to a range of complications, typically including pressure ulcers, facial edema, shoulder displacement, and peripheral nerve injuries [5-9]. While the association between obesity and increased complications due to PP is not unexpected, obesity is also a risk factor for more severe disease that necessitates prolonged PP and ultimately extends the length of ICU and hospital stays [10]. As of now, there have been no reports of enterocutaneous fistulas resulting from prolonged PP.

## Case presentation

**Figure 1:**
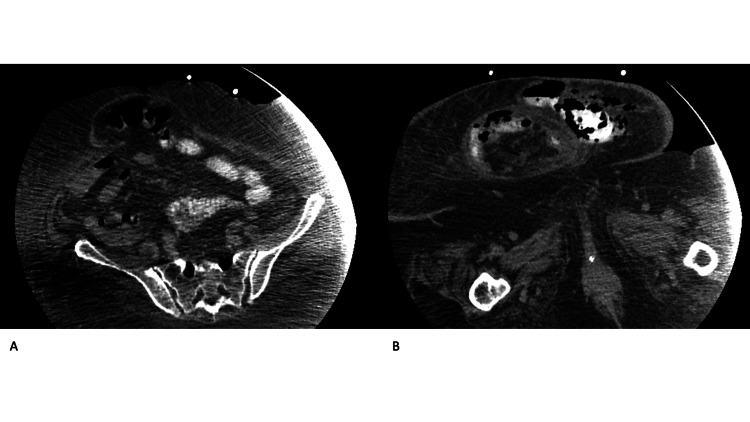
Computed tomography Α. Abdominal computed tomography showing a large periomphalic hernia with intestinal components, embedded in the adipose tissue of the abdominal wall. B. Presence of a second large abdominal wall hernia in the lower abdomen. There are signs of intestinal perforation in the abdominal wall (air bubbles in the adipose tissue), while a part of the intestine is in close proximity to the skin, indicating the presence of an enterocutaneous fistula.

We present the case of a 68-year-old woman with prolonged hospitalization due to COVID-19-related ARDS. Her medical history included morbid obesity (body mass index (BMI): 41 kg/m^2^) and arterial hypertension. In addition, she had undergone an uncomplicated low anterior rectal resection for colon cancer, with temporary ileostomy, four years ago. No other medical procedures were mentioned in her medical history. The patient presented to the hospital with respiratory failure due to COVID-19 and five days later, as respiratory failure worsened, she was intubated and transferred to the ICU. Upon ICU admission, the patient presented severe hypoxemia (PaO_2_/FiO_2_: 45 mmHg) and was set on PP. She received multiple sessions of proning (changing from prone to supine every 24 hours) during the first 16 days due to refractory hypoxemia (total hours in PP 210/384 (55% of ICU time)). Thereafter, the patient was prone for six to eight hours/day for the next seven days. Moreover, she presented hemodynamic instability upon admission, which required vasopressors (noradrenaline), due to septic shock from Enterococcus faecalis. A new episode of septic shock during the fifth ICU day (Klebsiella pneumoniae bloodstream infection) mandated escalation of vasopressor dose, while vasopressin was also added. The patient suffered multiple septic episodes, thereafter, and was vasopressor-dependent for most of the ICU time. On the 32nd ICU day, the patient presented signs of a new septic shock episode (new fever and hypotension warranting increased vasopressor doses). Gastroparesis was also present, thus parenteral nutrition was initiated. On clinical examination, a small blister was noticed on the abdominal wall. The patient was heavily sedated (under neuromuscular blockade as well), so abdominal pain and rebound tenderness could not be evaluated. Blood cultures drawn on the day of shock revealed the presence of three micro-organisms (Klebsiella pneumonia, Pseudomonas aeruginosa, and Enterococcus faecium). On the 36th ICU day, the blister was ruptured, and leakage of bowel contents was visible from the abdominal wall. An abdominal computed tomography (CT), with IV contrast, revealed the presence of an enterocutaneous fistula at the middle abdominal line, in continuum with an intestinal hernia (not previously known from the patient’s history) (Figure 1). The patient was transferred to the operating room where intestinal perforation was found; the involved intestinal segment and the abdominal wall including the fistula and the necrotic subcutaneous tissue were excised, and the skin was converged. The histopathologic examination revealed only normal bowel tissue and necrotic tissue. Postoperatively, the case was complicated by wound dehiscence, which was treated conservatively with antibiotics and the wound packaged with absorbent ribbon gauze. The patient gradually improved and was discharged from the ICU on the 55th day and from the hospital on the 75th day.

Outcome and follow-up

The patient was discharged from the hospital on the 75th day to a rehabilitation center due to ICU-acquired muscle weakness (ICU-AW), which was likely a result of the extended length of stay in the ICU, the severity of the critical illness, and sepsis-associated neuromyopathy. It is worth noting that corticosteroids, administered as a standard therapy for severe COVID-19 patients, as well as the SARS-CoV-2 infection itself, might have played an additional role in this condition. Furthermore, long-COVID-19 patients frequently report persistent fatigue and myalgia. She remained at the rehabilitation center for 60 days, and before her discharge, she regained the ability to stand up unassisted. Six months later, her health improved, walked approximately one kilometer every two days, and her BMI decreased to 30 kg/m².

## Discussion

In our case, we hypothesize that in this morbidly obese patient, the applied pressure, while on PP, caused hernias in intestinal necrosis and entrenched rupture in the adipose tissue of the abdominal wall, ultimately leading to skin erosion (necrosis) and enterocutaneous fistula. Prolonged need for vasopressors due to multiple septic shock episodes additionally contributed to regional bowel and skin necrosis. Heavy sedation and neuromuscular blocking agent use precluded the identification of signs of intestinal rupture in the abdominal wall, probably resulting in the last septic shock episode [11]. Critical illness malnutrition may have also played a role.

The implementation of PP in severe ARDS patients has been shown to improve oxygenation status, mitigate ventilator-induced lung injury, and most importantly, increase survival rates [2,3]. Consequently, PP is recommended by clinical practice guidelines for managing severe ARDS cases that last for more than 12 hours [12]. As the COVID-19 pandemic evolved, the American Association for Respiratory Care issued recommendations endorsing the use of PP in patients experiencing severe hypoxemia due to COVID-19 [13]. Recently, Kharat et al. reported that during the pandemic, PP was employed in nearly 70% of ICU-admitted patients, in contrast to pre-pandemic studies where PP was utilized in less than 20% of mechanically ventilated ARDS patients [14].

In our department, PP was employed in 80% of the 320 mechanically ventilated patients who were intubated over the last two years of the COVID-19 pandemic, with each session typically lasting 24-30 hours. The optimal duration for PP has not been definitively established. The landmark study on PP demonstrated a mortality benefit with 16-hour sessions in patients with severe ARDS [3]. More recently, sessions lasting 45.7 to 56.6 hours were feasible and did not show significant differences in the outcome or the rate of complications such as pressure ulcers [15].

In our clinical practice, we have adopted a standardized protocol for the use of PP in patients, based on the PROSEVA study [3]. Special care is taken with obese patients to minimize pressure on the abdominal wall, using additional pillows in the thorax and groin. So far, complications have been relatively minor, including facial edema, pressure ulcers (notably on the nose, lips, and forehead), and occasional dislodgement of nasogastric tubes. The occurrence of an enterocutaneous fistula was an unexpected and uncommon complication, not previously reported in such cases. Established causes of enterocutaneous fistulas encompass factors such as foreign bodies (or trauma-induced injuries), radiation, inflammation (e.g., Crohn's disease), infection (e.g., tuberculosis and actinomycosis), epithelialization, neoplasia, and distal obstruction; the acronym "FRIEND" delineates these etiologies [16]. Possibly, the addition of a skin examination when turning patients from supine to prone and vice versa must be included in these protocols. 

Regarding complications, the reported incidence varies widely, primarily contingent on the duration of prone sessions [4]. Many studies indicate pressure ulcer prevalence rates ranging from 44% to 77% [6-8]. Binda et al. reported a pressure injury prevalence of 32.2% related to PP, predominantly affecting the face [9]. Furthermore, they documented bleeding in 25% of cases, often occurring at the sites of the nose and mouth due to interactions with nasogastric and endotracheal tubes [9]. A study involving prolonged prone sessions of nearly three days (total duration of 123±83.6 hours) identified deep tissue pressure injuries and minor pressure wounds in 71.5% of patients [4]. More severe complications such as brachial plexus injuries, shoulder and hip contractures, and ocular issues like elevated intraocular pressure, and ischemic optic neuropathy have been reported in the context of PP during the COVID-19 pandemic [17-20].

Recently, a comprehensive review aimed to identify adverse events (AEs) linked to PP in mechanically ventilated adults with ARDS, along with strategies to mitigate these AEs. The review included 41 studies, 15 of which pertained to COVID-19 ARDS patients. Over 40 distinct AEs were identified, with the highest pooled occurrence rates observed for severe desaturation (37.9%), barotrauma (30.5%), pressure sores (29.7%), ventilation-associated pneumonia (28.2%), facial edema (16.7%), arrhythmia (15.4%), hypotension (10.2%), and peripheral nerve injuries (8.1%) [5].

To the best of our knowledge, this is the first reported case where prolonged PP resulted in skin, abdominal wall, and intestinal necrosis, culminating in the formation of an enterocutaneous fistula. Clearly, preexisting hernias, previously overlooked due to increased abdominal wall adipose tissue (as illustrated in Figure 1), played a fundamental role in this process by entrapping the intestine under pressure. The administration of vasopressors may have also exerted a significant influence. Despite our vigilant approach to proning obese patients, who are at an elevated risk for pressure ulcers, managing these patients, particularly those with COVID-19, remains inherently challenging. Detecting bowel sounds is challenging, and an ultrasound exam may not always be feasible, especially in cases with increased penetration depth. Furthermore, the unprecedented nature of the pandemic, which necessitated numerous patients to be placed in the PP simultaneously as was the case with our patient, may lead to unforeseen AEs.

## Conclusions

In conclusion, we report a patient with morbid obesity and severe Covid-19 ARDS, who received multiple sessions of prolonged proning, resulting in improvement in terms of oxygenation. An unpredictable complication of enterocutaneous fistula, in the setting of intestinal hernia, was observed and was successfully managed surgically. PP has been extensively used in COVID-19 ARDS, as it improves oxygenation and might also have an impact on survival. However, PP is not without complications and except for usual AEs, physicians should always be alert for the occurrence of unpredictable complications. 

PP has been proven to improve oxygenation, lung injury, and mainly survival in Severe ARDS patients. Prolonged PP has been extensively used in intubated COVID-19 ARDS patients demonstrating survival benefit. Adverse complications, though typically minor and reversible, are not uncommon, with facial edema and pressure ulcers being the most frequent. Severe complications are more likely, particularly during prolonged PP sessions in obese patients, which pose specific management challenges. Enterodermal complication associated with PP is a really rare complication; It must be considered in a patient with known or suspected intestinal hernias, in cases of extreme obesity and extended proning sessions. 
